# The Coordinated Development and Regulation Research on Public Health, Ecological Environment and Economic Development: Evidence from the Yellow River Basin of China

**DOI:** 10.3390/ijerph19116927

**Published:** 2022-06-06

**Authors:** Wei Wei, Chenggong Jin, Ying Han, Zhenhui Huang, Tong Niu, Jinkai Li

**Affiliations:** 1 Center for Energy Environment & Economy Research, School of Management, Zhengzhou University, Zhengzhou 450001, China; weiwei123@zzu.edu.cn (W.W.); jin13462702773@gmail.com (C.J.); hanna3639@163.com (Y.H.); 202022024010545@gs.zzu.edu.cn (Z.H.); 2 Research Center for Economic Development and Environment of the Yellow River Basin, Zhengzhou University, Zhengzhou 450001, China; 3 Yellow River Institute for Ecological Protection & Regionally Coordinated Development, Zhengzhou University, Zhengzhou 450001, China

**Keywords:** yellow river basin, public health, ecological environment, economic development, coupling coordination theory

## Abstract

The dual problems of the public crisis from the global epidemic and the deterioration of the ecological environment constrain the economic development in the Yellow River Basin. To promote the sustainable and balanced development in the Yellow River Basin, this paper takes public health, ecological environment, and economic development, as a whole, to study the coordinated development of the Yellow River Basin. Based on coupling coordinated theory, we use the SMI-P method to evaluate the coordinated development index of public health, the ecological environment, and economic development in the Yellow River Basin. Moreover, we use the coordinated regulation and obstacle factor diagnosis to identify the main influencing factors and design regulation methods to optimize the coordinated development index. The results found that (1), during the research period, there is spatiotemporal heterogeneity in the coordinated development level in the Yellow River Basin. From 2009 to 2019, the overall development index increased steadily, while the regional disparity in the coordinated development level was obvious. (2) The ecological environment indicators contribute more to the relevance and obstacle factors, such as the average concentration of fine particulate matter, per capita arable land area, afforestation area, etc. (3) After regulating the overall development level of the Yellow River Basin, we prove that Path 4, which comprehensively considers the relevance and obstacle factors, performs better.

## 1. Introduction

Since the outbreak of the global epidemic, COVID-19, there has been an increasing concern about public health issues [1,2,3]. Meanwhile, the deterioration of the ecological environment, caused by economic development, also has a great impact on public health, which restricts social development [4]. The Yellow River Basin is rich in grain, coal, and oil, which make it an important economic zone in China. To be specific, the regional GDP of Yellow River Basin accounts for more than 1/4 of the national total output value. Moreover, the Yellow River Basin is an important ecological barrier in northern China. However, the ecological environment of the Yellow River Basin is getting worse because of intensified human destruction and tremendous resource consumption [5,6]. It’s necessary to take a series of effective measures to improve the ecological conservation capacity and the restoration of ecological barrier of the Yellow River Basin. Furthermore, the Yellow River Basin flows through nine provinces, whose population accounts for about 1/3 of China. The public health in the Yellow River Basin is facing challenges due to environmental degradation and other issues. Therefore, increasing investment in public health and improving the level of public health can narrow the gap in people’s livelihood, maintain sufficient healthy labor, and guarantee food production and national security in the Yellow River Basin. In summary, the ecological environment, public health, and economic development of the Yellow River Basin are interconnected. Comprehensive treatment for the ecological environment can safeguard public health and propel the economic development of the Yellow River Basin. In turn, the safeguard of citizens’ health provides labor security for economic development, and economic development will also facilitate the infrastructure improvement to further enhance the level of public health, as well as the restoration and protection of the ecological environment.

Under the background of ecological protection and high-quality development of the Yellow River in China, we explore the synergistic relationship among public health, the ecological environment, and economic development, which aims to provide effective suggestions for the high-quality development of the Yellow River Basin. Existing studies have shown that there is a close and complex causal relationship among the economic development system, public health system, and the ecological environment system [7]. Relatively speaking, studies on the relationship of the three subsystems are more complex than mere dyads.

For the relationship between public health and economic development, the US Special Administrative Committee on Occupational and Environmental Medicine Health emphasized the impact of health on the economy from the perspective of health productivity and health care crisis [8]. Nichol et al., designed a retrospective cohort study and used multivariate models to analyze the experimental data, to assess the socioeconomic burden of the disease, from the perspective of an invasive disease [9]. Some scholars used the Granger-type causality test to empirically demonstrate the impact of public health on economic development from both macro and micro perspectives [10]. On the other hand, high-quality social and economic development promotes people’s income growth and improves public health indirectly. First, economic growth promotes adequate social health care facilities, which will improve the public health [11]. Second, economic growth brought by technological progress can improve the medical level and further promote public health [12]. Stenberg et al. used simulation modelling to assess the socioeconomic returns of health investments, and they demonstrated that investments in public health can significantly improve both public health and social returns [13]. On the contrary, economic recession will have an adverse impact on the public health level [14,15]. For the sake of long-term development, scholars have reviewed previous research findings, on public health and economics, to make recommendations for the coordinated development of the two subsystems [16,17].

In terms of the relationship between public health and the ecological environment, about 1.6 million deaths each year can be attributed to the unhealthy air, which accounts for about 17% of all deaths in China [18]. Grossman pioneered a healthy production model to describe the relationship between healthy input and output [19]. Cropper, Gerking, and Stanley improved the healthy production model and proposed a simple model of preventive health care and a health-oriented choice model, respectively, revealing that the key factors affecting public health are health care, genetics, the environment, lifestyle, etc. [20,21]. Based on empirical data, Wells and Evans explained the relationship between public health and the ecological living environment of citizens [22]. Given the existing research results, Koehler et al. proposed a conceptual framework to consider the impact of environmental decision-making on public health, further emphasizing that public health is not only related to the living environment but also to local transportation and energy [23]. Various resources in the ecological environment are closely related to the survival of human beings, and they also have a significant impact on the public health level. The important ecological resources, such as water resources [24], forest resources, [25], and air resources [26] are destroyed and polluted seriously, and the resulting public health problems have triggered extensive research in the field. In addition, some scholars have innovatively discussed public health from the perspective of food pesticide residues [27].

For the relationship between the ecological environment and economic development, existing studies verified that the inverted U-shaped relationship between economic growth and environmental degradation is based on the empirical framework of the Environmental Kuznets Curve (EKC) [28,29]. In recent studies, Hao et al. draw the similar conclusions of the inverted U-shaped relationship, when they studied the relationship between China’s ecological environment and economic development, by using the spatial lag model SLM and spatial panel data model, respectively [30,31]. On the other hand, the defects of the ecological environment will also restrict the economy development. For example, Saidi and Hammami found that energy consumption has a positive impact on economic growth, while carbon dioxide has a negative impact on economic growth based on synchronous equations [32]. In addition, some scholars use various models to study the impact of the ecological environment on the economy, such as the coupling coordination model. Shi T et al. measured the coupling coordination and spatial heterogeneity of economic development and the ecological environment in parts of China through Geographically and Temporally Weighted Regression (GTWR) [33]. Liu et al. used the coupling coordination model and Geographically Weighted Regression (GWR) to analyze the coupling coordination relationship between economic development and the ecological environment in the Yellow River Basin [34]. Recently, some scholars have used the coupling model to carry out research. Li et al. used an obstacle degree model, based on the coupling coordination model, to diagnose the obstacle factors affecting the coupling coordination [35]. Zhang et al. used the Tapio decoupling model and the STIR PAT model to analyze the economic output and water environmental pressure in the Yangtze River Basin, and they put forward relevant suggestions [36]. Some scholars use the environmental Impact-GDP-Technology (IGT) decoupling model to study the economic growth and energy consumption of developed and developing countries, indicating that the decoupling index of developed countries is better than the index of developing countries [37]. With further research on the relationship between the two, more and more scholars propose that environmental protection should be promoted by reducing the speed of economic growth [38,39].

“Coupling coordination” originates from the field of physics and refers to the close connection between each internal and external element of two or more systems. It is mainly used in the field of climate change. As the research moves along, coupling coordination has been introduced to measure the nonlinear interactions between the ecological environment and economic development, as well as public health and economic development. Liao et al. measured the degree of coupling and coordination between economic development and the ecological environment in the Beijing-Tianjin-Hebei region of China, and they made policy recommendations for local coordinated development [40]. Wu et al. studied the coordinated development level of China’s overall economic development and the ecological environment, and they conducted an empirical analysis of the spatial and temporal distribution of coordination in 31 provincial-level regions in China [41]. In addition, Zou et al. used the coupling coordination method to evaluate the coordinated development between economic development and public health in Sichuan, but they found that the level of coupling coordination was not ideal [42]. In conclusion, as for the relationship among public health, the ecological environment, and economic development, current studies are mostly discussing the relationship between two of them, while lacking the comprehensive researches on the three-subsystem relationship and the coordinated development path of the three subsystems for regulation. However, only by considering the correlation among the three, comprehensively, can we find out the certain problems existing in the coordinated development from an overall perspective. We comprehensively evaluate the coordinated development level of public health, the ecological environment, and economic development, and we systematically consider how to promote the overall coordination level and high-quality development in the Yellow River Basin.

## 2. Methodology

### 2.1. Quantitative Evaluation Method of Coordinated Development

The evaluation method of “single index quantification-multi-index synthesis-multi-criteria integration” (SMI−P) is usually used to evaluate the interaction and change between different systems [43]. We adopt the fuzzy analysis method to quantify the high-quality development indicators of public health in the Yellow River Basin to the [0,1]. According to the impact of indicators on the index of coordinated development, the indicators are divided into positive and negative indicators. The corresponding quantitative calculation formulas for indicators are positive Equation (1) and negative Equation (2). Given the regional characteristics and index properties, we determine each index node according to the selected quantification method. Among them: the average value of each indicator in the Yellow River Basin, over the years, is regarded as the passing value (*c*); the optimal value (*e*) represents the maximum value of the indicator increased by 10%; the worst value (*a*) is the minimum value of the indicator reduced by 10%; the worse value (*b*) represents the interpolation of *a* and *c*; the better value (*d*) represents the interpolation of *c* and *e*.

(1) $$SHD_{i}=\left\{ \begin{array}{l} 0\;\;\;\;\;\;\;\;\;\;\;\;\;\;\;\;\;\;\;\;\;\;\;\;\;\;\;\;\;\;\;\;\;\;\;\;\;\;\;\;\;\;\;\;(x_{i}\leq a_{i})\\ 0.3(\frac{x_{i}-a_{i}}{b_{i}-a_{i}})\;\;\;\;\;\;\;\;\;\;\;\;\;\;\;\;\;\;\;\;\;\;\;\;\;\;(a_{i}<x_{i}\leq b_{i})\\ 0.3+0.3(\frac{x_{i}-b_{i}}{c_{i}-b_{i}})\;\;\;\;\;\;\;\;\;\;\;\;\;\;\;\;\;(b_{i}<x_{i}\leq c_{i})\\ 0.6+0.2(\frac{x_{i}-c_{i}}{d_{i}-c_{i}})\;\;\;\;\;\;\;\;\;\;\;\;\;\;(c_{i}<x_{i}\leq d_{i})\\ 0.8+0.2(\frac{x_{i}-d_{i}}{e_{i}-d_{i}})\;\;\;\;\;\;\;\;\;\;\;\;\;\;(d_{i}<x_{i}\leq e_{i})\\ 1\;\;\;\;\;\;\;\;\;\;\;\;\;\;\;\;\;\;\;\;\;\;\;\;\;\;\;\;\;\;\;\;\;\;\;\;\;\;\;\;\;(e_{i}<x_{i}) \end{array}\right.$$

(2) $$SHD_{i}=\left\{ \begin{array}{l} 1\;\;\;\;\;\;\;\;\;\;\;\;\;\;\;\;\;\;\;\;\;\;\;\;\;\;\;\;\;\;\;\;\;\;(x_{i}\;\leq \;\;e_{i})\\ 0.8+0.2(\frac{d_{i}-x_{i}}{d_{i}-e_{i}})\;\;\;\;\;\;\;\;\;\;\;\;(e_{i}<\;x_{i}\;\leq \;d_{i})\\ 0.6+0.2(\frac{c_{i}-x_{i}}{c_{i}-d_{i}})\;\;\;\;\;\;\;\;\;\;\;\;\;(d_{i}<x_{i}\;\leq \;c_{i})\\ 0.3+0.3(\frac{b_{i}-x_{i}}{b_{i}-c_{i}})\;\;\;\;\;\;\;\;\;\;\;\;\;\;(c_{i}<x_{i}\leq \;b_{i})\\ 0.3(\frac{a_{i}-x_{i}}{a_{i}-b_{i}})\;\;\;\;\;\;\;\;\;\;\;\;\;\;\;\;\;\;\;\;(b_{i}<x_{i}\;\leq \;a_{i})\\ 0\;\;\;\;\;\;\;\;\;\;\;\;\;\;\;\;\;\;\;\;\;\;\;\;\;\;\;\;\;\;\;\;(a_{i}<x_{i}) \end{array}\right.$$

For the three subsystems of the ecological environment, economic development, and public health, we adopt the method of multi-index integration to evaluate the coordinated development index of the three subsystems comprehensively. The calculation equations are:

(3) $$EEDI(T)=\sum_{i=1}^{n_{1}}w_{i}SHD(Y^{i}(T))$$

(4) $$HQEDI(T)=\sum_{i=1}^{n_{2}}w_{i}SHD(Y^{i}(T))$$

(5) $$PHDI(T)=\sum_{i=1}^{n_{3}}w_{i}SHD_{3}(Y^{i}(T))$$

where T represents the year, *i* represents the evaluation indicator, and the variable $SHD(Y^{i}\left(T\right)$ represents the result of single-index quantization; EDI(T), HQEDI(T), and PHDI(T) represent the index of the ecological environment, economic development, and public health in the *T* year, respectively. $n_{1}$, $n_{2}$, and $n_{3}$ are the number of evaluation indicators of the ecological environment, economic development, and public health subsystem, respectively; $w_{i}$ is the weight of each indicator.

The Yellow River Basin Public Health High Quality Coordinated Development Index (EHP) is composed of three subsystem development indices, EEDI, HQEDI, and PHDI, by the following equation:

(6) $$EHP(T)=\beta_{1}EEDI(T)+\beta_{2}HQEDI(T)+\beta_{3}PHDI(T)$$

where $\beta_{1}$, $\beta_{2}$, and $\beta_{3}$ represent the system weights of the given EEDI(T), HQEDI(T), and PHDI(T), respectively, and $\beta_{1}=\beta_{2}=\beta_{3}=1/3$.

After calculating the coordinated development index of the public health, ecological environment, and economic development, the index is divided into seven levels, according to its value, as shown in Table 1.

### 2.2. Coordinated Identification

Coordinated identification makes quantitative analysis easier through the coordinated relationship between two or more parties, which can be used to identify the contribution of each indicator to the overall coordinated development index of the system. Modeling identification and non-modeling identification are two methods for coordinated identification. Particularly, the non-modeling identification method is used in the paper to identify the main influencing factors of the coordinated development of public health, the ecological environment, and economic development in the Yellow River Basin. The calculation process is as follows [44]:

- Determine the reference sequence and the comparison sequence, the dependent variable constitutes the reference sequence $x_{0}$, and the independent variable constitutes $x_{i}$.

$$x_{0}(k)=\{x_{0}(1),\;x_{0}(2),\;\ldots \;,\;x_{0}(n)\;(k=1\;,\;2\;,\;\ldots \;,\;n)$$

$$x_{i}(k)=\{x_{i}(1),\;x_{i}(2),\;\ldots \;,\;x_{i}(n)\}\;(k=1\;2\;,\;\ldots \;,\;m)$$

b. The data is dimensionless to obtain the sequences $x_{0}\prime$ and $x_{i}\prime$. The common methods are the mean value method and the initial value method, with the former used at this point.

(7) $$\Delta_{0i}(k)=\left|x_{0}\prime (k)-x_{i}\prime (k)\right|$$

c. Correlation degree $r(x_{0},x_{i})$ calculation.

(8) $$r(x_{0},\;x_{i})=\frac{\left({\mathrm{Min}}_{i}{\mathrm{Min}}_{k}\Delta_{0i}(k)+\xi {\mathrm{Max}}_{i}{\mathrm{Max}}_{k}\Delta_{0i}(k)\right)}{\left(\Delta_{0i}(k)+\xi {\mathrm{Max}}_{i}{\mathrm{Max}}_{k}\Delta_{0i}(k)\right)}$$

In the equation, ξ is the resolution coefficient. With the value range of (0,1), ξ generally takes 0.5. Moreover, $\Delta_{0i}\left(k\right)$ is a difference sequence.

d. Calculate the grey correlation degree $r_{0i}$.

(9) $$r_{0i}=\frac{1}{n}\sum_{k=1}^{n}r(x_{0}(k),\;\;\;\;\;x_{i}(k))$$

### 2.3. Obstacle Factors Diagnosis

The obstacle degree model is used to diagnose the obstacle factors, which can identify the main influencing factors on the overall coordination. The specific calculation processes are as follows [45]:

1. Calculate the factor contribution degree $F_{j}$ of the *j*th evaluation index:

(10) $$F_{j}=w_{j}w_{j}{}^{*}$$

where $w_{j}{}^{*}$ represents the weight of the subsystem to which the jth indicator belongs.

2. Calculate the deviation

(11) $$I_{j}=1-x_{ij}$$

3. Calculate the obstacle degree $P_{j}$ of each evaluation index:

(12) $$P_{j}=\frac{F_{j}I_{j}}{\sum_{j=1}^{n}F_{j}I_{j}}$$

We can obtain the obstacle degree of each index, in all provinces and regions, by using the same method to diagnose the obstacle factors.

### 2.4. Coordinated Regulation

Coordinated regulation aims to ameliorate the coordinated development index of the overall system by some reconciliation steps [43]. There are two methods for coordinated regulation: (1) adopt the optimal method of coordinated behavior set; (2) determine the minimum range of coordinated balance based on the coordinated balance optimization model. The first method is used for regulation in the paper.

## 3. System Construction and Data Sources

**Figure 1:**
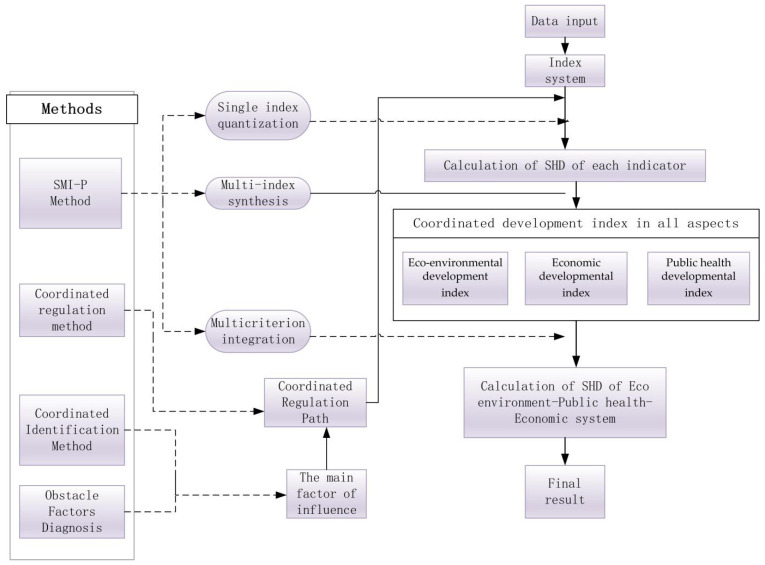
Research framework.

According to the existing research frameworks of coordinated development and regulation research [44], we establish the modified research framework, as shown in Figure 1. The specific research steps are summarized as follows.

First of all, we need an index system to evaluate the coordinated development index of public health, the ecological environment, and economic development in the Yellow River Basin. The existing research focuses, mainly, on the provincial and municipal levels [46,47]. Given the difficulty in prefecture data acquisition and the multiple research indicators in this paper, we research the Yellow River Basin, at the provincial level, based on the “Outline of Ecological Protection and High-quality Development Planning in the Yellow River Basin”. The public health, ecological environment, and economic development system is an open but complex system, and whether the index system is scientific or systematic will affect the evaluation effect. According to existing theoretical results [44], we construct the index system from three subsystems of ecological environment, economic development, and public health system to measure the coordinated development level of the Yellow River Basin. The existing research results, which have established two index systems of economic development and ecological development, are limited [48]. First, the original index system only considered the two dimensions of economic structure and resource consumption when measuring economic development. Based on the outline of the Yellow River strategic plan, this paper evaluates the regional economic system from the three dimensions of economic foundation, scientific and technological innovation, and opening to the outside world. The important indicators, such as the number of R&D personnel among 10,000 employees, the number of people engaged in scientific and technological activities, and the number of higher education graduates are included to measure regional economic innovation. Second, the original indicator system emphasized ecological space area, total resources, etc., while it ignored regional disparity in economy, population, and area. Therefore, we incorporate the per capita park green space, afforestation area, and per capita water consumption into the index system to reflect the ecological environment in the basin. Third, the original index system did not consider public health. The public health indicators can reflect the regional economic development level. Therefore, we take public health as one of the subsystems in the evaluation index system. In conclusion, the portfolio of the evaluation index system is critical, which should follow the principles of scientificity, systematicness, operability, availability, etc. To be specific, the determined evaluation index system includes three first-level indicators of the ecological environment, economic development, and public health, as well as 10 s-level indicators of the ecological environment pressure, health service and security, and economic foundation, in addition to 39 three-level indicators, which is shown in Table 2.

To facilitate the indicators’ labeling and selection, all indicators are coded by the XABC method. The index number starts with X, and A represents the subsystem (1 represents EEDI, 2 represents HQEDI, 3 represents PHDI), B represents the classification layer of the subsystem, and the last C represents the index number of the corresponding indicator. For example, X1101 represents the index 01 of the first-level classification layer of the ecological environment system. The specific codes are shown in Table 2.

Secondly, we determine the node value of each indicator based on its properties, such as a, b, c, d, and e, whose specific values are shown in Table 3. The SMI−P method is used to calculate the EHP by using Equations (1)–(6). Thirdly, using Equations (7)–(9) of coordinated identification, in combination with EHP, can calculate the contribution of each indicator to the overall development index of the Yellow River Basin. Fourthly, using the SHD, as well as Equations (10)–(12) can help calculate the obstacle degree of each indicator with regards to the overall coordinated development index of the Yellow River Basin. Finally, after coordinately regulating the quantitative index system of public health and high-quality development in the Yellow River Basin, the second step is repeated to obtain the regulated coordinated development index.

**Figure 2:**
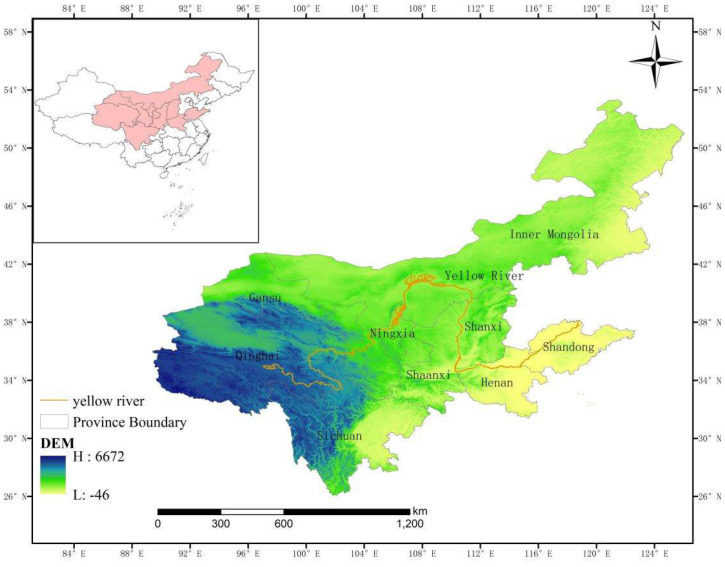
Yellow River Basin Map.

Figure 2 demonstrates the general situation of the Yellow River Basin. There is a close relationship between public health, the ecological environment, and economic development in the Yellow River Basin. We select the Yellow River Basin as the research object, and we analyze and evaluate the relationship between the overall public health, ecological environment, and economic development. The data for each indicator comes from “*China Statistical Yearbook*”, “*China Health Statistical Yearbook*”, “*China Environmental Statistical Yearbook*”, “*China Rural Poverty Inspection Report*”, “*China’s Statistical Bulletin of Outbound Investment*”, and the Statistical Yearbooks of the Yellow River Basin Provinces. Some missing data is complemented by means of adjacent years or linear interpolation.

## 4. Empirical Results and Discussion

### 4.1. Analysis and Evaluation of the Coordinated Development Index of Public Health, Ecological Environment, and Economic Development in the Yellow River Basin

According to the established evaluation index system for the coordinated development of the public health, ecological environment, and economic development in the Yellow River Basin, we use the SMI−P method to process and calculate the data. The overall *EEDI*, *HQEDI*, and *PHDI*, from 2009 to 2019, are obtained, and we get the overall *EHP* through Equation (6), as shown in Table 4. In addition, we also calculate the coordinated development index of the provinces, upstream, midstream, and downstream, as shown in Table 5. Finally, according to the classification criteria in Table 1, the coordinated development index of each region is classified into different grades, and the results are shown in Table 6.

#### 4.1.1. Analysis of the Overall Coordinated Development Index of the Yellow River Basin

Table 4 shows the overall coordinated development index of the Yellow River Basin from 2009 to 2019, which shows an upward trend. Specifically, the overall coordinated development index is from 0.352 to 0.486 in a decade, and the coordination level is from “less coordinated” in 2009 to “close to coordination” in 2011, and it remains stable in the subsequent stage. It demonstrates that the overall uncoordinated problem of high-quality public health development in the Yellow River Basin is relatively prominent, and there is much space for strengthening the connections between subsystems.

**Figure 3:**
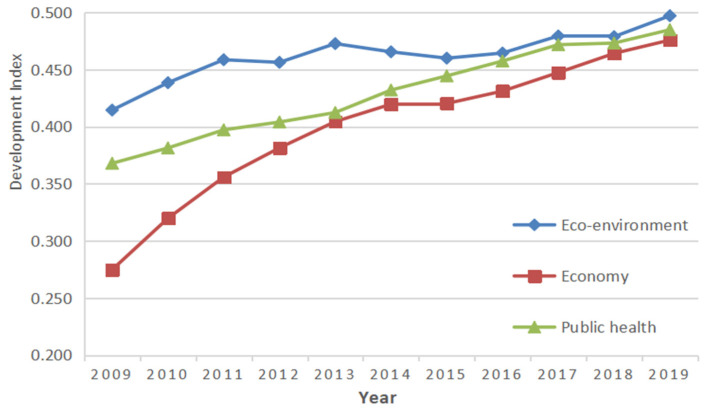
The overall subsystem development trend change diagram.

Compared to Table 4, the Figure 3 can directly demonstrate the evolutionary trend of the respective development indices of the Yellow River Basin subsystems. The ecological environment coordinated development index shows a trend of a wave-like rise [49], which can be attributed to the promotion of environmental protection awareness among people, the improvement of the environmental protection system, and the construction of large-scale basic environmental protection facilities. Taking 2012 as a key turning point, the ecological environment development index before 2012 was on a declining curve, which proves the rapid economic development in the basin led to the deterioration of the ecological environment [48,50]. While after 2012, the governance of ecological environmental protection, coordinated economic development, and the transformation and upgrading of heavily polluting enterprises were enhanced under the guidance of the “Twelfth Five-Year Plan” and the “Thirteenth Five-Year Plan”, as a result, the ecological environment system and economic development system appear with an upward trend again. The public health development index climbs steadily from 2009 to 2019, and there was a vigorous growth after the “Healthy China 2030” in 2016.

#### 4.1.2. Analysis of the Coordinated Development Index of the Provinces in the Yellow River Basin and the Upstream, Midstream and Downstream

**Figure 4:**
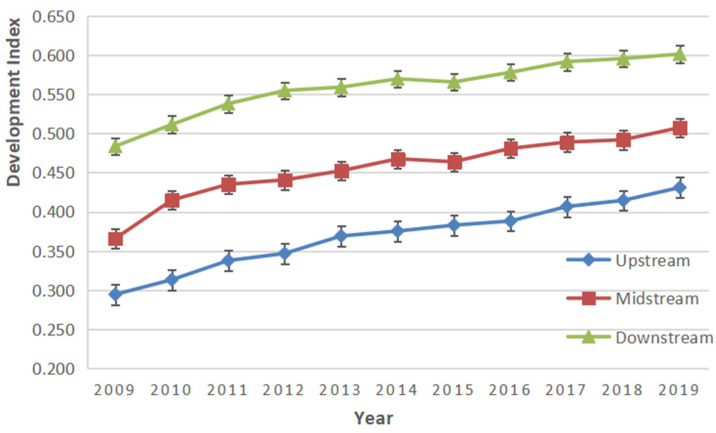
Coordinated development trend of upstream, midstream, and downstream.

Using the coordinated development index of the upstream, midstream, and downstream, from 2009 to 2019 in Table 5, its trend map can be drawn, as shown in Figure 4. In combination with Table 5 and Table 6, we can have a comprehensive analysis of the coordinated development index of the provinces in the Yellow River Basin and the upstream, midstream, and downstream.

From the perspective of each province, the development index of public health, the ecological environment, and economic development in nine provinces is mainly concentrated as 0.1~0.7, involving four levels of basically uncoordinated, less uncoordinated, close to coordination, and more coordinated. Specially, the coordinated levels of Qinghai, Gansu, and Ningxia are less coordinated, and Inner Mongolia, Shaanxi, Shanxi, and Henan are close to coordination. Furthermore, Sichuan has developed from close to coordination to more coordinated, and Shandong is more coordinated, which reaches the highest level of coordination. From the perspective of upstream, midstream, and downstream, the coordinated development level of the downstream is the highest, which is close to coordination, while the level of the midstream has changed from less coordinated to close to coordination. However, the level of the upstream is less uncoordinated, which demonstrates the massive gap among upstream, midstream, and downstream. The coordinated status of each subsection of the basin is consistent with the actual situation. There are, indeed, certain gaps and barriers in the coordinated development index between provinces, as well as the upstream, midstream, and downstream [51,52].

On the whole, the coordinated development index of each province and upstream, midstream, and downstream have shown an upward trend, which indicates that the coordinated development of the three subsystems of the Yellow River Basin shows a positive rising trend in the future.

#### 4.1.3. Spatial Evolution Analysis of the Coordinated Development Index of Public Health, Ecological Environment and Economic Development in the Yellow River Basin

**Figure 5:**
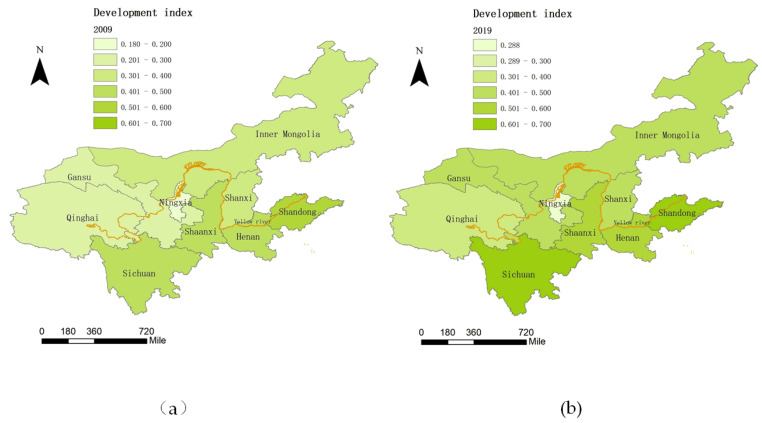
Coordination index spatial distribution: (**a**) is the distribution of 2009 and (**b**) is the distribution of 2019.

We used the coordinated development index of each province in Table 5 to draw the spatial distribution of the coordinated development index of each province in the Yellow River Basin in 2009 and 2019, as shown in Figure 5. From the perspective of the overall spatial distribution, the coordinated development index of the Yellow River Basin has improved significantly from 2009 to 2019, which shows a progressively increasing distribution pattern from west to east [34]. The developed transportation and policy dividends in the downstream of the Yellow River Basin make the prior economic development level and public health index of the provinces in the downstream [53]. In addition, although desertification in the upstream has been effectively curbed, the low vegetation coverage still constrains the comprehensive development of the upstream of the Yellow River. This distribution pattern is consistent with the actual situation in the Yellow River Basin. From the perspective of provinces, the coordinated development indices of each province differ greatly. Compared with neighboring provinces, Sichuan and Shandong have relatively higher coordinated development indices. Due to the superior geographical location at the estuary of the Yellow River, Shandong performs well in economy development, the ecological environment, living conditions, and public health. Sichuan is the passage of both the Yellow River and the Yangtze River. With the unique geographical advantage, Sichuan has a higher comprehensive development level, especially with the ecological environment. The development index of Ningxia is always the bottom level, but its increment of development index has little difference with other regions. Restricted by a small population and inland locations, the comprehensive development levels of Xinjiang and Tibet are greatly affected by economic development and the ecological environment. The ideal state is to break down administrative barriers and play some leading role of high-level regions to promote the coordinated development of surrounding regions.

### 4.2. Coordinated Identification of Public Health, Ecological Environment and Economic Development in the Yellow River Basin

#### 4.2.1. The Relationship between Public Health, Ecological Environment and Economic Development in the Yellow River Basin

Based on the calculation of SMI−P method, we carry out the coordinated identification. Take the time series of EHP as the reference series ($x_{0}$(*k*)) and the SHD of each index as the comparison series ($x_{i}\left(k\right)$) to calculate the grey correlation degree, whose results are shown in Table 7. Due to the large number of indicators, each province only lists the first eight indicators with larger values, which are recorded as key indicators. The growing grey correlation degree indicates the increasingly high level of contribution to the overall development.

The degree of contribution to the three-subsystem coordinated development, of each indicator in the nine provinces, can be seen in Table 7. On the whole, the key indicators of each province are mainly concentrated on the economic subsystem and the public health subsystem, indicating that the economic and public health subsystems play a dominant role in the overall development index of the Yellow River Basin. In terms of specific indicators, there are eight indicators that appear no less than four times in the key indicators. The ecological environment subsystem supplies three of the eight indicators: average concentration of fine particulate matter, per capita arable land, and effective utilization coefficient of farmland irrigation water, respectively. The economic subsystem supplies two of the eight indicators; they are the number of people engaged in scientific and technological activities and the number of higher education graduates. The public health subsystem also supplies three of the eight indicators: the number of beds in health care institutions, the number of travel agencies, and the number of medical institutions are the corresponding indicators. The eight indicators are, basically, even distributed, indicating that the three subsystems must be developed in a coordinated manner to continuously improve the overall coordinated development level in the Yellow River Basin, thus making the Yellow River Basin more coordinated as a whole.

#### 4.2.2. The Yellow River Basin Obstacles of Public Health, Ecological Environment and Economic Development

We use Obstacle Factors Diagnosis to calculate the obstacle degree of each index of the nine provinces in the Yellow River Basin, in 2019, and select the eight indicators with higher obstacle degree rankings in each province as the main obstacle factors, which is shown in Table 8.

Table 8 quantitatively shows the restrictive effect of each indicator on the coordinated development of public health, the ecological environment, and economic development in the Yellow River Basin. The larger obstacle degree is the stronger restrictive function. On the whole, economic development subsystem indicators appear more frequently than public health subsystems and ecological environment subsystems. The result is in line with the fact that economic growth promotes the perfection of public facilities, which subsequently improves the public health level and the ecological environment. In terms of specific indicators, some indicators are universal, including the total sewage discharge, afforestation area, number of R&D personnel among 10,000 employees, natural population growth rate, government health spending as a percentage of health spending, and the number of days that the air quality reaches the second level and above [35]. Among them, the total amount of sewage discharge and the afforestation area belong to the ecological environment subsystem. The sewage discharge from polluting enterprises in the Yellow River Basin for economic growth has actually affected the environment. It is crucial to take measures to reduce pollution. Given the collateral effect of the coordination level, the afforestation expansion is another urgent task. The number of R&D personnel among the 10,000 employees is an economic indicator, which reflects insufficient innovation capability in economy development. The natural population growth rate, the government health spending as a percentage of health spending, and the number of days that the air quality reaches the second level and above are the indicators of the public health subsystem, The air quality reaching the second level and above means that the Air Quality Index (AQI) is no more than 100 [54]. Only when the air quality reaches the second level and above can it meet the living standard of “healthy air quality”. The indicators of the public health subsystem reflect several realistic problems: for example, the declined natural population growth in China, the inadequate obligations for public health of the government, and the harm to citizens’ health caused by the ecological environment deterioration.

### 4.3. Coordinated Regulation of Public Health, Ecological Environment and Economic Development in the Yellow River Basin

**Figure 6:**
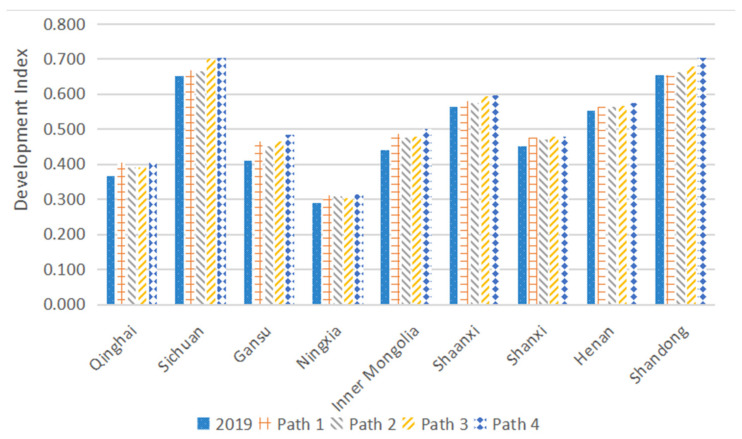
Dynamic changes in the development index of each province.

According to the coordinated development results calculated by the SMI−P method, it’s not difficult to find that the overall coordinated development level of the Yellow River Basin is close to coordination. To obtain the optimal adjustment method, we adjust each indicator of the Yellow River Basin through the optimal set of four coordination behaviors, which are shown in Table 9:

The original data are regulated through the coordinated behavior regulation path, and then, the coordination development index of each path is calculated by the SMI−P method. The calculation results are shown in Table 10, and the dynamic change chart of the development index in each province is shown in Figure 6.

On the whole, the results in Figure 6 and Table 10 imply the coordinated development index of each province in each path. Particularly, the coordinated development index growth of each path in Sichuan, Gansu, Inner Mongolia, and Shandong is relatively significant.

In terms of the specific paths, Path 4 has the best coordinated regulation effect from the holistic watershed perspective, which integrally considers the correlation and constraints. The overall coordinated development index increases by 0.42. Moreover, the adjustment effect of other paths, from high to low, is Path 3, Path 1, and Path 2. From the adjustment effect of each province, the Path 4 is still the best one, while Path 1, Path 2, and Path 3 have their own advantages in the adjustment effect when applied to different provinces. In summary, the optimal adjustment method for the optimal set of coordinated regulatory behaviors should be comprehensively considering the correlation and constraints that affect the overall development index. For the indicator’s adjustment, it is necessary to comprehensively consider its own relevance and obstacle factors, and finally, Path 4 is used as the optimal adjustment method in the paper.

## 5. Discussion

Existing studies have already developed several methods to quantitatively evaluate the overall coordinated development of the Yellow River Basin. Among them, the coupling coordination method is widely used. The coupling coordination method can measure the close relationship between systems and the influence of one thing on the others [55]. Table 11 lists some studies that used the coupling coordination method and did some innovations to evaluate the coordinated development level in the Yellow River Basin. As for the research systems, the evaluation of the two systems of the Yellow River Basin is the choice of more scholars. Based on the coupling coordination method, this paper uses the SMI−P method to evaluate the three systems of public health, the ecological environment, and economic development in the Yellow River Basin, and it adopts coordinated identification and obstacle factor diagnosis to identify and regulate the main influencing factors. Not coincidentally, the research period in this paper is basically consistent with some existing studies. The main reason is the long period of about ten years can help to comprehensively reflect the overall development level and the long-standing problems of the Yellow River Basin. It found that most of the existing studies focus on the evaluation of coupling coordination and the identification of the main influencing factors, but there are a few explorations on the regulation of the main influencing factors. Therefore, we complement the previous research properly. Based on the evaluation results, we identify the main influencing factors and design four methods to explore the optimal method. On the whole, the overall coordinated index shows a slow upward trend with spatiotemporal heterogeneity [56], which is similar to the existing research conclusions. In addition, we also found that the overall coordinated development index of the Yellow River Basin is not only affected by the common factors, such as population size and per capita natural growth rate [35,57], but also by other specific factors, such as the number of days that air quality reaches the second standard and above, the number of medical institutions, number of higher education graduates, etc. We also reach the conclusion that the method which comprehensively considers the relevance and obstacle factors for regulation has the best overall regulation effect.

At present, the coupling coordination degree method is mainly used to evaluate the coordination development level [58]. Compared with the existing coupling coordination research method, we use the SMI−P method to measure the coordination development level in the yellow river basin. The two research methods have similarities and differences. The difference is mainly reflected in the different data normalization methods. The SMI−P method uses the segmentation fuzzy membership analysis method to process the data, while the coupling coordination method uses the normalization method. The same points are reflected in: ① They all use the entropy weight method to weigh the indicators. ② Both the SMI−P method and the coupling coordination method have a guiding role for reality. For example, Li and Yi use the coupling coordination method to evaluate the economy, society, and the environment of nine central cities in China [59]. Zuo used the SMI−P method to evaluate the relationship between humans and water in the Tarim River in China [60].

In addition, we considered the research scope. We chose the entire province of the watershed as the study area, instead of the prefecture-level cities, because it is difficult to form a relatively complete index system due to the inaccessible data in prefecture-level cities.

## 6. Conclusions and Policy Implications

Based on the coordinated development of the three subsystems of the ecological environment, economy, and public health, we research the nine provinces in the Yellow River Basin and construct the evaluation system for the public health, ecological environment, and economic development as a whole. In addition, the correlation degree and obstacle degree of the system indicators that affect its coordinated development index are calculated. Furthermore, the coordinated regulation method is adopted to regulate the index system. Based on the above processes, we draw some conclusions:

(1) The coordinated development index of the public health, ecological environment, and economic development, during the study period, shows an increasing trend [34,49]. The ecological environment index (*EEDI*) has a higher base value but the slowest growth rate compared with the ecological environment index and economic index [48]. The base value of the public health index (*PHDI*) and the growth rate are in the middle level. The index of each subsystem in the Yellow River Basin tends to be consistent. However, the overall development index level is not prominent, and each subsystem still has a huge space for ameliorating the development level.
(2) During the study period from 2009 to 2019, the overall coordinated development index kept growing steadily from 0.352 to 0.486. The level of coordinated development also has promoted from less coordinated to close to coordination. When discussing the coordinated development level of the upstream, midstream, and downstream, the upstream comes last, followed by the midstream, with the downstream topping the table [34]. As for the coordinated development level of the nine provinces, Shandong and Sichuan are at the peak levels, which are close to coordination [48]. While Ningxia and Qinghai are ranked the worst performers, which is still at a basically uncoordinated level. Other provinces are basically close to coordination. On the whole, the certain gap and barrier in the coordinated development index among the basins cannot be ignored, as well as the nine provinces. [53].
(3) In terms of correlation, the indicators that have a general impact on the overall coordinated development index of the Yellow River Basin, including the average concentration of fine particulate matter, the per capita arable land, the effective utilization coefficient of farmland irrigation water, the number of people engaged in scientific and technological activities, the number of higher education graduates, the number of beds in health care institutions, the number of travel agencies, and the number of medical institutions [57]. In terms of the obstacle degree, the indicators that have a general restrictive effect on the overall coordinated development index of the Yellow River Basin, including the total sewage discharge, afforestation area, number of R&D personnel among 10,000 employees, natural population growth rate, government health spending as a percentage of health spending, and the number of days that the air quality reaches the second level and above [35]. According to the optimal method of coordinated behavior set, we construct the index system and found that regulation Path 4 is the optimal regulation method, which comprehensively considers the relevance and obstacle factors.

Based on the above research results and conclusions, the following policy recommendations are proposed for the high-quality development in the Yellow River Basin.

(1) In terms of the ecological environment, strengthen the intensity of sewage treatment, increase investment in sewage treatment facilities, ameliorate sewage treatment standards to reduce pollution of ecological water, and ensure water safety for residents. In addition, treating agricultural equipment, improving the utilization rate of agricultural irrigation water, and reducing the use of agricultural fertilizers and pesticides can reduce the burden on land and make land sustainable. Moreover, promote the transformation and upgrading of heavily polluted enterprises, use clean energy, develop new technologies, and improve the efficiency of resource utilization and the recycling rate of waste, thereby reducing solid particulate matter and harmful gas emissions and improving air quality. Furthermore, strengthen afforestation, increase the coverage rate of forests and wetlands in the Yellow River Basin, and promote the restoration of the ecological environment on both sides of the Yellow River and the prevention and control of river basin pollution to enhance the environmental carrying capacity and the ability to restore the ecological environment, and the ecological barrier function of the Yellow River Basin can be stably played.
(2) In terms of economic development, consider the actual situation of the region, optimize the industrial structure, attach importance to technological innovation, increase investment in research and development, use the actual policy to introduce talents in order to transform the local industry into technology-intensive industries and enhance the local economic creativity. Furthermore, policies guide the increase in the share of the tertiary industry structure, and they increase the disposable income of residents to stimulate economic growth to achieve the purpose of stimulating residents’ consumption and economic growth. Besides, regions should strengthen cooperation with neighbors, so provinces with better economic development can play a leading role in promoting the development of surrounding regions, accelerate economic development in poor regions, and narrow economic regional differences. At the same time of economic development, properly balancing the relationship with other industries enables the formation of a new regional economic pattern with complementary advantages and characteristic development, which realizes the sustainable development of the regional economy.
(3) In terms of public health, raise the concept to guide population growth, improve the working welfare of medical personnel, and strengthen policy subsidies for drugs to reduce the degree of aging, improve the motivation of medical staff, and reduce the personal health consumption expenditure of residents. In addition, increase care for the elderly, strictly monitor drinking water resources, improve urban infrastructure and emergency facilities in provinces, and prevent public health crises caused by emergencies, so the lives, health, and safety of residents can be guaranteed, and the happiness and satisfaction of living can be improved. Furthermore, developing fitness and entertainment venues, travel agencies, and other venues for physical exercise and spiritual entertainment can improve the health of residents and achieve the goal of the Healthy China.
(4) Promote the comprehensive management of the public health, ecological environment and economy subsystems. The economy can drive the improvement of ecological environment protection and public health, while the high quality of the ecological environment and public health can also promote economic development. Moreover, by breaking administrative barriers, promoting regional open cooperation, market cooperation, brand development, and benefit sharing, establish cooperative and mutual aid relationships and, ultimately, realize the overall high-quality development of the Yellow River Basin

From an application point of view, the above suggestions may be helpful for policy design. In addition, the scientific and reasonable policies can improve the level of coordinated development of public health, the ecological environment, and economy in the Yellow River Basin, and they promote the high-quality development of public health in the Yellow River Basin, which is in line with our research purposes.

Academically, the paper can effectively make up for the relative insufficiency of the current studies on the regulation method of the main factors affecting the overall coordinated development index, and it can also provide auxiliary reference for further research by other scholars. In the future, we will conduct more detailed research on the Yellow River flowing through prefecture-level cities. In addition, this study assigns one-third of the weight to each subsystem of public health, the ecological environment, and economic development, and future research will assign weight to each system in a more rational manner.

## Figures and Tables

**Table 1:** The grades of the coordination index of public health and high-quality development in the Yellow River Basin.

Coordination Level	Coordinate Grading	The Value Range of *EHP*
fully coordinated	VII	1
basic coordination	VI	[0.8,1)
more coordinated	V	[0.6,0.8)
close to collaboration	IV	[0.4,0.6)
less coordinated	III	[0.2,0.4)
basically uncoordinated	II	(0,0.2)
totally uncoordinated	I	0

**Table 2:** Quantitative index system for high-quality development of public health in the Yellow River Basin.

Subsystem	Classification Layer	Indicator Layer	Explanation	Unit	Index Number
Ecological environment	Ecological environment pressure	Fertilizer (Pesticide) Application Amount	The amount of fertilizers (pesticides) actually used in agricultural production	104 tons	X1101
		Per capita water consumption	Total water supply/population	m^3^/capita	X1102
		Total sewage discharge	The amount of sewage discharged from the sewage outlet	104 m^3^	X1103
		General industrial solid waste discharge	The amount of solid waste discharged outside the pollution prevention and control facilities	104 tons	X1104
		Average concentration of fine particulate matter	Average concentration of particulate matter less than 2.5 microns in diameter	μg·m^−3^	X1105
	Ecological environment	Per capita water resources	total water resources/total population	m^3^/capita	X1201
		Per capita park green space	Total park area/total population	104 m^2^/capita	X1202
		Development and utilization of water resources	Total water consumption × 100%/total water resources	%	X1203
		Per capita arable land	Total arable land area/total population	104 m^2^/capita	X1204
	Ecological environment response	Investment in pollution control as a percentage of GDP/%	Pollution treatment cost × 100%/GDP	%	X1301
		Afforestation area	Afforestation Area	108 m^2^	X1302
		Harmless treatment rate of domestic waste	Amount of garbage treated in a harmless manner × 100%/total amount of garbage	%	X1303
		Sewage treatment rate	sewage treatment volume × 100%/total sewage discharge	%	X1304
		Effective utilization coefficient of farmland irrigation water	The actual effective water use of farmland × 100%/total water consumption of farmland	%	X1305
		Soil erosion control area	The total area of soil erosion under control	104 km^2^	X1306
Economic development	Economic base	GDP per capita	GDP/total population	yuan	X2101
		Per capita disposable income	Income that everyone can use without limit	yuan	X2102
		Total retail sales of social consumption	The total volume of social consumer goods transactions	109 yuan	X2103
		The proportion of the tertiary industry	tertiary industry output value × 100%/GDP	%	X2104
	Science and education innovation	Number of R&D personnel among 10,000 employees	Total R&D employees × 10,000/total population	people	X2201
		Number of people engaged in scientific and technological activities	Number of people working in scientific and technological activities	people	X2202
		R&D spending intensity	R&D investment × 100%/total output	%	X2203
		Number of higher education graduates	Number of higher education graduates	people	X2204
	Opening to the outside world	Foreign direct investment	Amount invested by foreign businessmen	104 dollars	X2301
		Import and export volume	Total amount of goods actually imported and exported	104 dollars	X2302
		Degree of external dependence	Total import and export × 100%/GDP	%	X2303
Public health	level of health	Natural population growth rate	the number of natural population increasing × 100%/average total population	%	X3101
		Maternal mortality ratio	Total Maternal Deaths /the number of population increasing	1/103	X3102
		Aging proportion	Number of people over 65 years old × 100%/total population	%	X3103
	Health services and security	Personal hygiene expenditure as a percentage of health spending	Personal hygiene expenditure × 100%/total hygiene expenditure	%	X3201
		Government health spending as a percentage of health spending	Government health expenditure × 100%/total health expenditure	%	X3202
		Number of health technicians	Number of people working in health technology	people	X3203
		Number of beds in health care facilities	Total number of beds in medical institutions × 1000/total population	Piece/103	X3204
	Healthy environment	The number of days that the air quality reaches the second level and above	Number of days per year to achieve healthy air quality standards	day	X3301
		The proportion of surface water quality reaching or better than Class III water body	Number of water bodies with water quality reaching or better than Class III × 100%/total number of water bodies	%	X3302
	Health industry	Number of units of fitness, leisure and entertainment activities	Number of units of fitness, leisure and entertainment activities	\	X3401
		Number of travel agencies	Number of travel agencies	\	X3402
		Number of Aged Care Institutions	Number of Aged Care Institutions	\	X3403
		Number of medical institutions	Number of medical institutions	\	X3404

**Table 3:** The properties of each indicator of the quantitative index system for high-quality development of public health.

Indicator Layer	Unit	Index Number	*a*	*b*	*c*	*d*	*e*	Indicator Direction
Fertilizer (Pesticide) Application Amount	10^4^ tons	X1101	787.71	508.806	229.901	118.537	7.173	−
Per capita water consumption	m^3^/capita	X1102	1278.53	857.583	436.636	292.388	148.14	−
Total sewage discharge	10^4^ m^3^	X1103	375,011.12	242,215.677	109,420.234	60,124.517	10,828.8	−
General industrial solid waste discharge	10^4^ tons	X1104	40,233.27	27,278.754	14,324.238	7791.354	1258.47	−
Average concentration of fine particulate matter	μg·m^−3^	X1105	71.242	49.105	26.968	16.56	6.151	−
Per capita water resources	m^3^/capita	X1201	121.95	1302.133	2482.317	11,639.238	20,796.16	+
Per capita park green space	10^4^ m^2^/capita	X1202	7.191	10.158	13.125	18.14	23.155	+
Development and utilization of water resources	%	X1203	943.45	528.591	113.731	58.086	2.442	−
Per capita arable land	10^4^ m^2^/capita	X1204	0.675	1.351	2.028	4.094	6.16	+
Investment in pollution control as a percentage of GDP/%	%	X1301	0.468	1.106	1.745	3.089	4.433	+
Afforestation area	10^8^ m^2^	X1302	0.704	7.822	14.941	37.952	60.963	+
Harmless treatment rate of domestic waste	%	X1303	29.133	58.456	87.779	98.862	109.945	+
Sewage treatment rate	%	X1304	38.007	61.866	85.726	96.631	107.536	+
Effective utilization coefficient of farmland irrigation water	%	X1305	0.365	0.446	0.526	0.618	0.711	+
Soil erosion control area	10^4^ km^2^	X1306	0.009	0.147	0.285	0.539	0.794	+
GDP per capita	yuan	X2101	11,521.8	25,104.698	38,687.596	58,202.948	77,718.3	+
Per capita disposable income	yuan	X2102	5627.7	11,049.086	16,470.472	25,613.586	34,756.7	+
Total retail sales of social consumption	10^9^ yuan	X2103	272.571	3790.124	7307.676	19,741.987	32,176.298	+
The proportion of the tertiary industry	%	X2104	1.538	20.321	39.104	49.867	60.63	+
Number of R&D personnel among 10,000 employees	people	X2201	17.829	28.819	39.809	63.823	87.836	+
Number of people engaged in scientific and technological activities	people	X2202	3607.2	39,922.544	76,237.889	224,451.794	372,665.7	+
R&D spending intensity	%	X2203	0.432	0.853	1.273	1.963	2.653	+
Number of higher education graduates	people	X2204	13,194	99,731.475	186,268.949	419,504.475	652,740	+
Foreign direct investment	10^4^ dollars	X2301	3920.4	231,892.715	459,865.03	1,358,332.315	2,256,799.6	+
Import and export volume	10^4^ dollars	X2302	108,140.4	2,339,995.346	4,571,850.292	18,581,580.35	32,591,310.4	+
Degree of external dependence	%	X2303	1.501	5.937	10.374	23.66	36.945	+
Natural population growth rate	%	X3101	2.079	3.809	5.538	8.731	11.924	+
Maternal mortality ratio	1/10^3^	X3102	50.71	34.541	18.372	12.066	5.76	−
Aging proportion	%	X3103	18.437	14.022	9.608	7.267	4.926	−
Personal hygiene expenditure as a percentage of health spending	%	X3201	52.878	43.261	33.644	27.446	21.249	−
Government health spending as a percentage of health spending	%	X3202	19.62	26.265	32.909	43.828	54.747	+
Number of health technicians	people	X3203	21,096	141,220.232	261,344.465	560,933.932	860,523.4	+
Number of beds in health care facilities	Piece/10^3^	X3204	17,207.1	127,639.934	238,072.768	602,101.384	966,130	+
The number of days that the air quality reaches the second level and above	day	X3301	106.65	182.576	258.502	321.201	383.9	+
The proportion of surface water quality reaching or better than Class III water body	%	X3302	9	36.468	63.936	85.978	108.02	+
Number of units of fitness, leisure and entertainment activities	\	X3401	1.8	116.456	231.111	1117.656	2004.2	+
Number of travel agencies	\	X3402	76.5	452.093	827.687	1860.343	2893	+
Number of Aged Care Institutions	\	X3403	31.5	550.866	1070.232	2410.066	3749.9	+
Number of medical institutions	\	X3404	1412.1	14,646.474	27,880.848	60,006.774	92,132.7	+

Note: *c* represents the average value of each indicator over the years, *e* represents the maximum value of the indicator increased by 10%, *a* represents minimum value of the indicator reduced by 10%, *b* represents the interpolation of *a* and *c*, and *d* represents the interpolation of *c* and *e*.

**Table 4:** The overall coordinated development index from 2009 to 2019.

Subsystem	2009	2010	2011	2012	2013	2014	2015	2016	2017	2018	2019
*EEDI*	0.415	0.438	0.459	0.456	0.473	0.465	0.460	0.464	0.479	0.479	0.497
*HQEDI*	0.275	0.320	0.356	0.381	0.404	0.420	0.420	0.431	0.447	0.464	0.476
*PHDI*	0.368	0.381	0.397	0.404	0.412	0.432	0.444	0.457	0.472	0.473	0.485
*EHP*	0.352	0.380	0.404	0.414	0.430	0.439	0.442	0.451	0.466	0.472	0.486
Coordinated level	III	III	IV	IV	IV	IV	IV	IV	IV	IV	IV

Note: we obtain the *EEDI*, *HQEDI*, and *PHDI* through Equations (1)–(5), and the *EHP* is calculated through Equation (6).

**Table 5:** Coordinated development index of provinces and upstream, midstream, and downstream of the Yellow River Basin from 2009 to 2019.

Area	2009	2010	2011	2012	2013	2014	2015	2016	2017	2018	2019
Qinghai	0.227	0.226	0.258	0.259	0.287	0.304	0.295	0.311	0.320	0.337	0.367
Sichuan	0.490	0.519	0.549	0.567	0.575	0.595	0.604	0.626	0.653	0.643	0.650
Gansu	0.249	0.266	0.283	0.305	0.326	0.329	0.351	0.339	0.368	0.381	0.411
Ningxia	0.180	0.204	0.212	0.203	0.232	0.229	0.241	0.236	0.251	0.276	0.288
Inner Mongolia	0.326	0.350	0.386	0.401	0.426	0.419	0.423	0.429	0.441	0.436	0.440
Shaanxi	0.404	0.447	0.468	0.475	0.493	0.514	0.515	0.527	0.549	0.540	0.564
Shanxi	0.327	0.383	0.402	0.407	0.412	0.422	0.413	0.435	0.429	0.444	0.451
Henan	0.407	0.437	0.469	0.494	0.502	0.519	0.513	0.526	0.542	0.544	0.551
Shandong	0.560	0.586	0.607	0.616	0.616	0.621	0.619	0.630	0.642	0.647	0.652
Upstream	0.294	0.313	0.338	0.347	0.369	0.375	0.383	0.388	0.407	0.415	0.431
Midstream	0.366	0.415	0.435	0.441	0.452	0.468	0.464	0.481	0.489	0.492	0.508
Downstream	0.484	0.511	0.538	0.555	0.559	0.570	0.566	0.578	0.592	0.596	0.602

Note: the indices of each province, upstream, midstream, and downstream are obtained by Equations (1)–(6).

**Table 6:** Coordinated development levels of provinces and upstream, midstream, and downstream of the Yellow River Basin from 2009 to 2019.

Area	2009	2010	2011	2012	2013	2014	2015	2016	2017	2018	2019
Qinghai	III	III	III	III	III	III	III	III	III	III	III
Sichuan	IV	IV	IV	IV	IV	IV	V	V	V	V	V
Gansu	III	III	III	III	III	III	III	III	III	III	IV
Ningxia	II	III	III	III	III	III	III	III	III	III	III
Inner Mongolia	III	III	III	IV	IV	IV	IV	IV	IV	IV	IV
Shaanxi	IV	IV	IV	IV	IV	IV	IV	IV	IV	IV	IV
Shanxi	III	III	IV	IV	IV	IV	IV	IV	IV	IV	IV
Henan	IV	IV	IV	IV	IV	IV	IV	IV	IV	IV	IV
Shandong	IV	IV	V	V	V	V	V	V	V	V	V
Upstream	III	III	III	III	III	III	III	III	IV	IV	IV
Midstream	III	IV	IV	IV	IV	IV	IV	IV	IV	IV	IV
Downstream	IV	IV	IV	IV	IV	IV	IV	IV	IV	IV	V

Note: the result is obtained by using Table 4 and Table 5.

**Table 7:** Calculation results of the correlation degree.

Area	1		2		3		4		5		6		7		8	
	x	$r_{0i}$	x	$r_{0i}$	x	$r_{0i}$	x	$r_{0i}$	x	$r_{0i}$	x	$r_{0i}$	x	$r_{0i}$	x	$r_{0i}$
Qinghai	X1102	0.971	X3201	0.958	X3202	0.956	X1105	0.951	X3302	0.949	X1204	0.944	X1203	0.942	X1101	0.942
Sichuan	X3204	0.970	X3203	0.967	X3402	0.943	X2204	0.937	X1303	0.936	X2202	0.935	X2203	0.913	X2103	0.898
Gansu	X1305	0.993	X1204	0.964	X2204	0.957	X2202	0.957	X1102	0.954	X1105	0.950	X2203	0.946	X2104	0.944
Ningxia	X1202	0.977	X2104	0.973	X1301	0.959	X3404	0.958	X1204	0.949	X1105	0.946	X1101	0.939	X1104	0.937
Inner Mongolia	X3402	0.966	X1303	0.965	X1306	0.950	X3403	0.949	X1204	0.948	X2204	0.947	X2104	0.943	X1105	0.915
Shaanxi	X2201	0.945	X1305	0.944	X1105	0.940	X2204	0.939	X3402	0.931	X3302	0.924	X3102	0.912	X2203	0.909
Shanxi	X1305	0.981	X3404	0.964	X1304	0.957	X2204	0.951	X3402	0.946	X1306	0.945	X3204	0.943	X1105	0.933
Henan	X3203	0.976	X3204	0.975	X3404	0.968	X2204	0.965	X1304	0.962	X2202	0.962	X3102	0.955	X1305	0.955
Shandong	X2202	0.979	X3203	0.971	X3404	0.968	X2301	0.959	X1304	0.957	X2203	0.949	X3204	0.948	X1305	0.946

Note: The results are calculated by using the *EHP* and *SHD* already obtained, as well as Equations (7)–(9).

**Table 8:** Index barriers of provinces in the Yellow River Basin.

Area	1		2		3		4		5		6		7		8	
	x	$P_{j}$	x	$P_{j}$	x	$P_{j}$	x	$P_{j}$	x	$P_{j}$	x	$P_{j}$	x	$P_{j}$	x	$P_{j}$
Qinghai	X2104	39.8	X3102	27.9	X3401	24.6	X2203	23.6	X3203	22.6	X2103	22.0	X2204	21.4	X2201	20.9
Sichuan	X1301	20.8	X1302	18.5	X1305	18.4	X1103	18.3	X3103	16.2	X1204	15.3	X3101	13.3	X3202	13.3
Gansu	X2101	24.8	X2301	22.4	X2201	20.2	X3204	19.0	X2202	18.7	X2102	17.9	X2103	16.5	X2302	16.0
Ningxia	X1203	38.6	X1102	27.8	X3402	23.9	X3401	22.3	X3203	22.2	X2103	21.1	X1302	20.6	X2204	20.5
Inner Mongolia	X1102	22.5	X2203	20.7	X1104	19.5	X2201	17.2	X2301	16.5	X3101	16.5	X1302	15.0	X3404	14.6
Shaanxi	X1202	17.8	X3301	15.9	X1204	15.4	X3202	14.1	X1101	13.2	X2302	12.7	X1304	11.8	X1103	11.7
Shanxi	X3302	26.6	X1104	23.5	X2301	18.6	X1201	15.6	X2101	15.5	X3404	14.9	X1202	14.5	X3101	14.4
Henan	X1101	29.5	X1105	24.6	X1302	19.7	X1306	17.8	X3301	17.4	X1301	17.4	X1103	16.6	X3302	16.5
Shandong	X1105	28.9	X1103	24.6	X1101	19.5	X3302	19.3	X3103	18.7	X3202	18.2	X1306	17.2	X3301	16.7

Note: the results are calculated by using the *SHD* already obtained and Equations (10)–(12).

**Table 9:** The coordinated regulation path.

Path	Explanation
**Path 1**	The increment of all indicators adjusts to 1x the 2009–2019 increase.
**Path 2**	The increment of key indicators adjusts to double the 2009–2019 increase, The increment of other indicators adjusts by 0.8 times.
**Path 3**	The increment of major obstacle factors adjusts to double the 2009–2019 increase. The increment of other indicators adjusts by 0.8 times.
**Path 4**	Comprehensively consider the relevance and major obstacle factors to determine the increase multiple.

Note: key indicators and major obstacle factors represent top 8 indicators in relevance analysis and obstacle analysis, respectively.

**Table 10:** The development index of each province in the set of regulatory behaviors.

Area	2019	Path 1	Path 2	Path 3	Path 4
Qinghai	0.367	0.404	0.390	0.392	0.408
Sichuan	0.650	0.667	0.663	0.699	0.702
Gansu	0.411	0.464	0.450	0.465	0.483
Ningxia	0.288	0.310	0.309	0.303	0.312
Inner Mongolia	0.440	0.488	0.476	0.478	0.500
Shaanxi	0.564	0.581	0.574	0.593	0.595
Shanxi	0.451	0.476	0.471	0.478	0.479
Henan	0.551	0.564	0.563	0.565	0.574
Shandong	0.652	0.653	0.662	0.678	0.703

**Table 11:** Existing studies using the coupling coordination method in the Yellow River Basin.

Paper Authors	Research Period	Number of Systems	Methods	Main Conclusions
Zhao Y.; Hou P et al. [48]	2000–2018	2	coupling coordination model; evaluation method; coupling degree model.	The economic development index rose steadily, the ecological status index rose first and then fell; The degree of coupling slowly increased and then decrease;
Liu K; Qiao Y et al. [34]	2008–2017	2	coupling coordination model; geographical weighted regression.	The coupling coordination economic development and ecological environment showed regional heterogeneity; The coupling coordination degree is affected by population si1ze, openness and advanced industrial structure, etc.
Li H; Jiang Z et al. [35]	2010–2017	2	coupling coordination model; obstacle degree model	The coupling coordination social economic and resource environment showed an overall upward trend; The nine obstacle factors include natural growth rate of population, per capita green area of parks and so on
Qiu M; Yang Z et al. [57]	2008–2018	2	grey relationship and decoupling model	The urbanization level and ecological security level show an overall upward trend; There is a strong decoupling effect between them; The future ecological security will be more restrictive to the urbanization.

## Data Availability

Not applicable.
